# Chitosan Coating Inhibits the Growth of *Listeria monocytogenes* and Extends the Shelf Life of Vacuum-Packed Pork Loins at 4 °C

**DOI:** 10.3390/foods7100155

**Published:** 2018-09-25

**Authors:** Annalisa Serio, Clemencia Chaves-López, Giampiero Sacchetti, Chiara Rossi, Antonello Paparella

**Affiliations:** Faculty of Bioscience and Technology for Food, Agriculture and Environment, University of Teramo, via R. Balzarini 1, Teramo TE 64100, Italy; aserio@unite.it (A.S.); cchaveslopez@unite.it (C.C.-L.); gsacchetti@unite.it (G.S.); crossi@unite.it (C.R.)

**Keywords:** chitosan, *Listeria monocytogenes*, meat

## Abstract

Chitosan coating is a promising method for food preservation. This study aims to fill the data gap regarding the application of chitosan (1% and 2%) on vacuum-packed fresh pork stored at 4 °C for 28 days, with particular attention on the effect on *Listeria monocytogenes*, inoculated as a cocktail of three strains. Chitosan at both concentrations was able to significantly reduce *L. monocytogenes* counts by more than 1.5 Log CFU/g with respect to control; moreover, it inhibited the growth of mesophilic bacteria and was particularly effective on lactic acid bacteria and yeasts. The beneficial effects on shelf life were demonstrated by both panel test and pH evolution. In fact, panellists did not detect any sensory difference between samples treated with 1% chitosan and control up to 14 days of storage, while after 28 days, a pungent flesh odour was perceived in control samples only. Interestingly, at a_w_ values of fresh meat (0.984), the chitosan coating exhibited a liquid behaviour, with a dynamic viscosity of 229.4 ± 4.2 mPa/s. Chitosan coating applied on vacuum-packed pork loins contained *L. monocytogenes* growth and improved the microbiological characteristics of the product, with a beneficial effect on product shelf life.

## 1. Introduction

Fresh meat is a rich nutrient matrix that is highly prone to spoilage caused by different microorganisms, with subsequent biochemical and enzymatic deteriorations. In meats, vacuum-packaging and modified atmosphere are traditionally used to control microbial growth, improve safety, and delay spoilage [1]. Vacuum-packaging, which is generally more economical than modified atmosphere, is frequently used for primary cuts, while modified atmosphere packaging (MAP) is more suitable for retail displays of meat [2]. Furthermore, vacuum-packaging is frequently used in restaurants for temporary storage of meat slices. The exclusion of oxygen in vacuum-packaging inhibits aerobic bacteria, such as *Pseudomonas* species [2], allowing the growth of anaerobic and facultative anaerobic bacteria. Indeed, vacuum-packaging of chilled meat does not inhibit the development of *Listeria monocytogenes*, the presence of which, in meat products, is a particular food safety concern as it can cause listeriosis with serious effects, especially in elderly adults and immune-compromised people, causing meningitis, and in pregnant women, spontaneous abortion or stillborn babies [3]. An increase in the notified incidence rate of listeriosis in Europe has been reported during the years 2008–2015, with meat and meat products being often responsible of outbreaks [4]; in particular, this Gram-positive rod has the highest scores of risk for pork consumers [5]. 

Novel strategies have gained attention as a means to naturally control the growth of pathogenic and spoilage organisms. In fact, the increasing demand of consumers for better quality and improved freshness of food products has given rise to the development and implementation of edible films or antimicrobial dips on the surface of the product. Edible films or coatings are defined as continuous matrices that can be prepared from proteins, polysaccharides, and lipids [6], the application of which helps to maintain product quality, enhance sensory properties, and improve product safety. Chitosan (ß-(1,4)-2-amino-2-deoxy-d-glucopyranose) is a modified, natural carbohydrate polymer derived by deacetylation of chitin, which is the second most abundant biopolymer in nature next to cellulose, found in the exoskeleton of crustaceans, fungal cell walls, and other biological materials [7]. During the past several decades, chitosan has received increased attention for its commercial applications in the food industry, including food preservation, formation of edible biodegradable film, recovery of proteins from wastewater, and clarification of fruit juice. Furthermore, chitosan exhibits the potential to be used as a food supplement with antitumor, antiulcer, antiuricemic, and hypocholesterolaemic properties [8,9,10]. The biological safety of chitosan has been demonstrated and it is regarded as a safe food additive at a daily dose up to 3 g [11]. Due to its biodegradability, biocompatibility, atoxicity, and antimicrobial activity, chitosan shows wide possibilities of application in food bio-preservation [12,13]. 

Several studies have been performed on the application of chitosan on pork, particularly on minced meat [14], pork patties or burgers [15,16], slices [17], and stewed meat [18]. However, to the best of our knowledge, no information is available on the effect of chitosan on *L. monocytogenes* development in vacuum-packed pork under refrigeration. This bacterium is not inhibited by vacuum-packaging and is psychrotrophic; therefore, it is able to multiply during refrigeration, causing real concerns for the consumers safety. 

In the light of these considerations, the aim of this study was to investigate the antimicrobial activity of chitosan coating against *L. monocytogenes* and some microbial groups common in meat in vacuum-packed fresh pork stored at 4 °C for 28 days and also to determine some physical properties of the coating to better understand its behaviour. The temperature of 4 °C was selected as reference refrigeration temperature, while 28 days was chosen as the length of the commercial shelf life of this kind of products.

## 2. Materials and Methods

### 2.1. Microbial Strains and Growth Conditions

*L. monocytogenes* type strain ATCC 19114 (serotype 4a) and strains 95986 (serotype 1/2c) and 58712 (serotype 1/2c), previously isolated from deli meat sandwiches and pork ribs, respectively, and belonging to the collection of the Faculty of Bioscience and Technology for Food, Agriculture and Environment, University of Teramo were used in this study. The strains were stored in Microbank at −80 °C; to restore the viability of the strains, they were cultivated in two subsequent steps in Brain Hearth Infusion (BHI) Broth (Oxoid-Thermo Fisher Scientific, Rodano, Italy) at 37 °C for 24 h.

### 2.2. Preparation of Chitosan Solutions

Practical-grade chitosan from crab shells (Sigma-Aldrich, Milan, Italy), with less than 10% moisture and 75–85% deacetylation degree, was used. Chitosan powder was dissolved in an aqueous solution of glacial acetic acid 1%, as previously reported [12], to a final concentration of 1% and 2%. The solutions thus prepared were sterilized at 121 °C for 15 min and stored at 4 °C. All solutions were adjusted to a pH of 5.6.

### 2.3. Samples Treatment for the Evaluation of the Antimicrobial Activity of Chitosan on Pork Loins

The experimental work was focused on the evaluation of antimicrobial activity of chitosan against *L. monocytogenes* on samples of commercial pork loins. The tests were carried out by using a cocktail of the three strains (ATCC 19144, 95986, 58712), with a total count of approximately 5 × 10^5^ CFU/mL. Cells were harvested by centrifugation at 9300× *g* for 5 min (Eppendorf centrifuge 5415D) and washed three times with phosphate buffer saline (PBS) 50 mM, pH 7.4. The inoculum was spectrophotometrically standardized (Lambda Bio 20, Perkin Elmer, Boston, MA, USA) to O.D.620 values of 0.08–0.1, then diluted with sterile PBS to obtain a number of cells around 5 × 10^5^ cells/mL [18]. The number of cells was checked on BHI agar plates and incubated at 37 °C for 48 h.

Cubes of pork loin with a weight of about 3 g were aseptically prepared, and then the samples were flame sterilised on the surface for 30 s and stored in sterile Petri dishes to facilitate the subsequent operation of inoculation and drying. One millilitre of standardized inoculum (about 5 × 10^5^ UFC/mL) was inoculated on all the meat cubes, except for the negative controls (samples without inoculation), then left to dry for about 10 min under a biosafety cabinet (class II). 

A preliminary trial was performed to evaluate a possible contribution of acetic acid to the inhibitory activity of chitosan. Together with the inoculated controls, samples dipped with 1% chitosan and samples dipped (for 30 s) with 1% acetic acid were also prepared and left to dry for 1 h. 

In the main trial, together with the inoculated controls, two series of samples were dipped respectively in 1% and 2% chitosan solutions for 30 s and left to dry for 1 h. 

In both cases, the absorption of the chitosan solutions was determined by weighing the sample before and after dipping. Once dried, the samples were vacuum-packed (VM18, Orved srl, Musile di Piave, Italy), 100% vacuum for a total of 35–40 s, by using 3-mil polyamide-(nylon)-polyethylene vacuum pouch (Alpak Food Equipment, Portland, OR, USA), and the samples were stored at 4 °C for 28 days. Two different series of samples were prepared and analysed.

### 2.4. Microbiological Analyses

In the preliminary trial, microbial counts were analysed at time T0 (0 days) before treatment, T1 (1 day), T7 (7 days), and T14 (14 days) of refrigerated storage, as described below.

In the main trial, microbiological analyses were performed at time T0 (0 days) before treatment, T1 (1 day), T7 (7 days), T14 (14 days), T21 (21 days), and T28 (28 days) of storage at 4 °C. 

Samples were homogenized for 90 s in a Stomacher (Lab-Blender 400, PBI, Milan, Italy), and serial dilutions were prepared in sterile saline. The following microbial parameters were determined:

1. Mesophilic aerobic count on Plate Count Agar (PCA) (Oxoid), incubated at 30 °C for 48 h;
2. *L monocytogenes* in Listeria Agar acc. to Ottaviani & Agosti (ALOA) (Biolife Italiana), at 37 °C for 48 h;
3. Lactic acid bacteria (LAB) in MRS Agar (Oxoid), incubated at 30 °C for 48–72 h;
4. Coliforms were enumerated on Violet Red Bile Agar (VRBA) (Oxoid), at 37 °C for 24 h;
5. Yeasts were determined on Sabouraud Dextrose Agar (Oxoid), incubated at 25 °C for 72 h.

### 2.5. Physicochemical Analysis

For each meat sample, pH and water activity were analysed as meat quality indicators that were able to affect microbial spoilage. Meat samples were diluted 1:1 in deionized water and homogenized by Ultra-Turrax (T25 basic, IKA-Werke, Staufen, Germany) before pH measurement (MP 220 Mettler Toledo). Water activity was measured by AquaLab Series 3 (Decagon Devices, Pullman, WA, USA). Three repetitions for each measure were performed.

### 2.6. Sensory Analysis

Uninoculated samples were analysed by a triangle test [19] to evaluate three samples, where one was different from the other two. Samples treated with chitosan 1% and untreated samples were compared. The panel was made of 10 trained judges (25–50 years old). Panellists were invited to smell and taste the samples at time 1, 7, and 14 days, while at days 21 and 28, they were only requested to smell the samples to avoid potential microbiological risks. Judges were asked to identify the different samples. The triangle test was extended by applying a directional variation that required the assessors to identify the sample with higher odour intensity, and assessors were asked to indicate which sample presented the highest odour intensity. The analysis was performed as already described [17]. In detail, the pork samples were cooked in a ventilated electric oven Air-o-steam Combi 6GN (Electrolux, Stockholm Sweden) for 7 min to reach 70 °C at the core. Samples were then coded with three digits number and served in plastic dishes in a random order, and each test was performed twice in the same day. 

### 2.7. Determination of the Rheological Behaviour of Chitosan Film

Chitosan film was applied on Petri dishes, dried in the same conditions used for meat samples, and eventually allowed to equilibrate at a_w_ values of 0.984, corresponding to the lowest a_w_ value of chitosan-coated pork samples, for 7 days when it reached the a_w_ value of 0.984 and a liquid state.

Viscosity of chitosan solutions equilibrated at a_w_ 0.984 was thus measured at 25 °C with a falling-sphere viscometer type Thermo C (Haake, Karlsruhe, Germany). The measurements were carried out by using a boron silicate glass sphere with a density of 2.224 g/cm^3^ and K of 0.08112 mPa·s·cm^3^/g·s. The falling time of the sphere was measured with a stopwatch in correspondence with the passage between the two rings of reference of the viscometer in the step (A and B). The dynamic viscosity (mPa·s) was calculated using the following equation:

η = K (ρ1 − ρ2)·t(1)

where K = constant of the sphere in mPa·s·cm^3^/g·s. ρ1 = density of the sphere in g/cm^3^, ρ2 = liquid density in g/cm^3^, and t = time of fall of the sphere in s.

The density of the solution was measured at 25 °C with a hydrostatic balance Gibertini (Milan, Italy).

### 2.8. Mechanical Properties of Chitosan Coating

Chitosan coating was applied to sheets of gelatine to standardize the physical medium. The application of coating was the same used for meat samples. Following coating, the coated sheets were equilibrated to a_w_ 0.984 (obtained with 0.1 N KOH) and subsequently analysed. The uncoated sheets (reference) were equilibrated under the same conditions. The analysis of the mechanical properties of the sheets was conducted by an Instron Universal Testing Machine mod. 5542 dynamometer (Wycombe, UK), equipped with a load cell maximum of 0.5 kN. A compression-shear-extrusion test of 2.5 g of sample was carried out by using a cell Allo-Kramer operating with the following parameters: speed rate of 50 mm/min, sampling rate of 10 points/s, total stroke of 25 mm, and stroke extrusion of 10 mm. Compression modulus and force corresponding to the first peak cutting were the mechanical parameters considered.

### 2.9. Statistical Analysis

Analyses were run in triplicate. Textural analysis was run on five replicates. Means and standard deviations were calculated. The data obtained were subjected to ANOVA and Tukey’s HSD post hoc test was applied at *p* < 0.05 using Statistica 8.0 Software (Statsoft, Tulsa, OK, USA).

The statistical significance of triangle tests (discrimination among samples) was verified by comparing the total number of responses and the number of correct responses with the values reported in unilateral *p* = 1/3 probability tables. The statistical significance of the triangle directional tests was verified by comparing the number of correct responses in the first triangle test and the number of responses that referred to the most frequently selected odd sample in the directional test with the values reported in bilateral *p* = 1/2 probability tables.

## 3. Results and Discussion

### 3.1. Chitosan Effect on L. monocytogenes and on Meat Microbiota

The evaluation of the antimicrobial effects of chitosan against *L. monocytogenes* was carried out in accordance with European guidelines produced by the European Reference Laboratory ANSES [20], using a cocktail of one type strain and two strains isolated from meat products. Differently from ANSES guidelines, inoculum count was 5 × 10^5^ CFU/mL in order to assess the potential decrease of the inoculum during the experimental period. 

As the antimicrobial activity of acetic acid is well known, and the acid was used in a quantity of 1% to obtain the dipping solution of chitosan, a preliminary trial was performed in order to evaluate a possible contribution of this organic acid to the inhibitory activity of chitosan. Results are presented in Figure S1 (Supplementary Materials). The chitosan showed stronger inhibitory activity than acetic acid alone. In particular, the acetic acid was generally able to reduce the microbial counts after 1 day of refrigerated storage, but then they increased over time and were comparable to untreated samples after 14 days at 4 °C. Therefore, the inhibitory activity of chitosan was probably enhanced by the presence of acetic acid in its formulation, but the contribution of the acetic acid was negligible with respect to the action exerted by chitosan, in different ways depending on the microorganisms.

**Figure 1:**
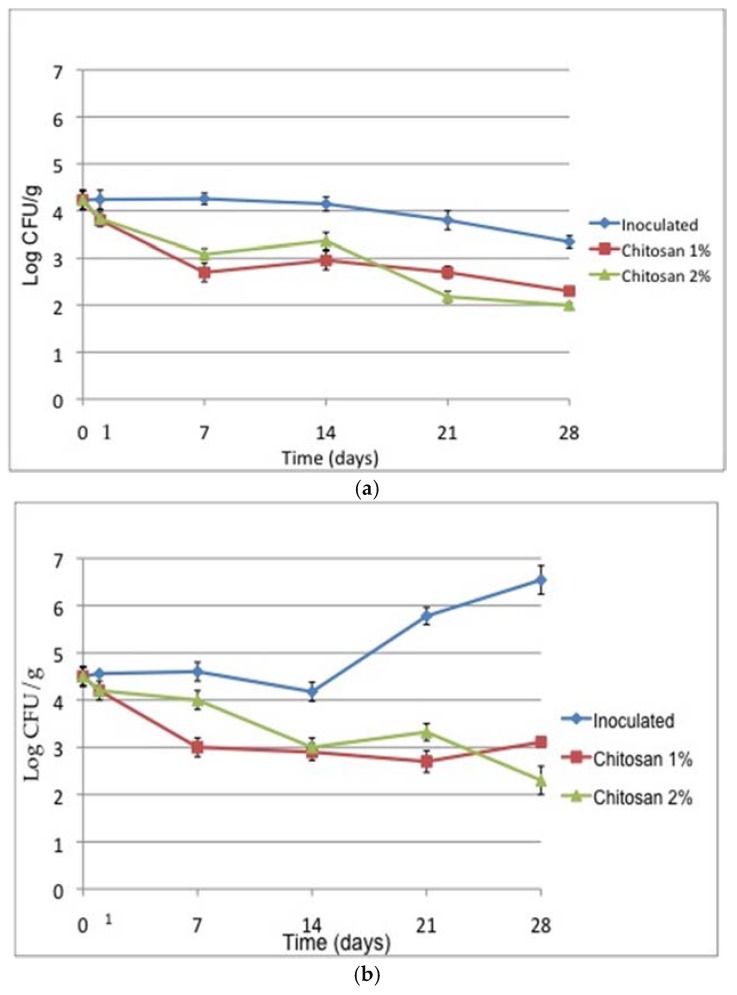

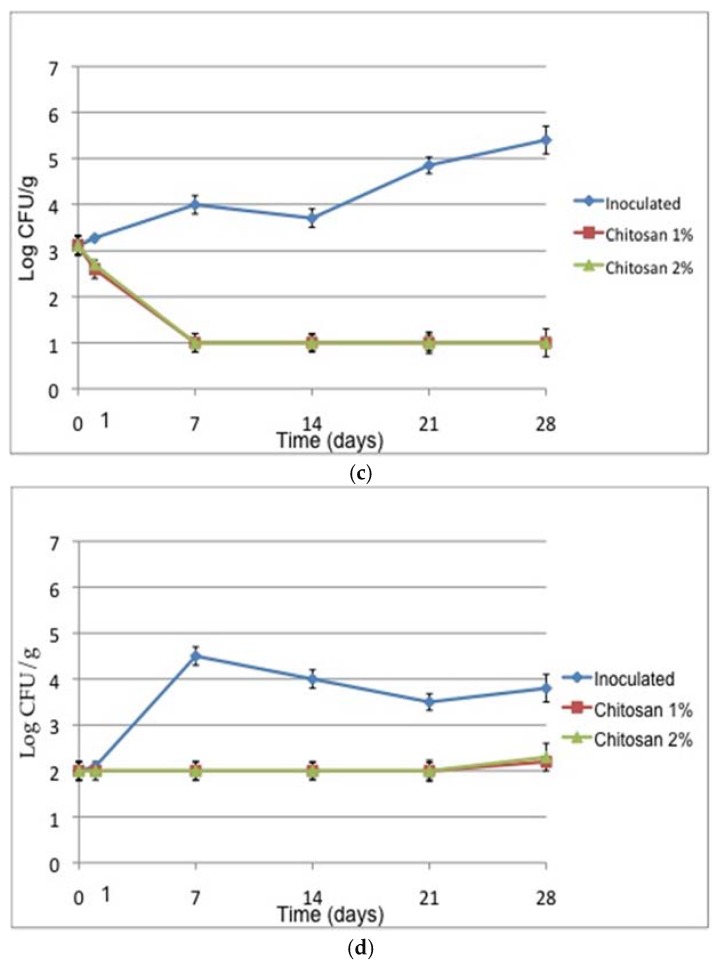
Evolution of the microbiota of vacuum-packed pork loin samples inoculated with *Listeria monocytogenes* (inoculated) after treatment with chitosan 1% or chitosan 2% over a 28-day period at 4 °C. (**a**) *L. monocytogenes*; (**b**) mesophilic aerobic count; (**c**) lactic acid bacteria; (**d**) yeasts.

Regarding the main trial, Figure 1 shows the behaviour of *L. monocytogenes* during refrigerated storage of vacuum-packed pork samples treated with 1% and 2% chitosan compared with the samples inoculated with the cocktail of strains in the absence of chitosan (positive control). Only 1 day after treatment, the count of the pathogen was reduced with respect to control, and after 7 days of refrigerated storage, a reduction of about 1.5 Log for the samples treated with 1% and 2% of chitosan was appreciable. A coating of chitosan 1% applied on pork meat under modified atmosphere reduced the *L. monocytogenes* count of about 2 Log CFU/g after 2 days of refrigerated storage, with a bacteriostatic effect up to 15 days of storage [21]. For food safety objectives, it is necessary to reduce the risk as much as possible. Therefore, to boost the effect of chitosan against foodborne pathogens, chitosan coatings are usually combined with essential oils or other natural bio-preservatives. 

In the inoculated sample, the *L. monocytogenes* count decreased over time but still remained around 3.4 Log CFU/g after 28 days of storage. The reduction could be attributed to the combined effect of vacuum-packaging and growth of lactic acid bacteria, which are known to exert antagonistic activity on *L. monocytogenes* [22]. Chitosan at both concentrations was significantly effective (*p* < 0.05) in reducing *L. monocytogenes* count until 28 days of storage, with 2% showing a better inhibitory activity in the long term compared to the positive control. 

To get a more complete framework of the microbial community of the samples, the evolution of mesophilic aerobic bacteria and lactic acid bacteria was determined. The solution of 1% chitosan appeared to be the most effective in containing mesophilic aerobic count up to 21 days, while after 28 days, 2% chitosan appeared to be more effective (Figure 1b). 

In the control, despite vacuum and refrigeration, mesophilic aerobic count increased steadily after a settling found at day 14, probably due to the selection of microbial groups better adapted to the conditions of the samples. At the end of the storage period, the count was below an arbitrary maximum acceptable limit of 7.0 Log CFU/g [17], and the effect of chitosan in containing the growth was highly significant (*p* < 0.01). Other authors proved the efficacy of chitosan in controlling the microbial growth in minced pork meat [14]. Moreover, the effect of 1% chitosan in reducing the aerobic count in pork sausages stored at 4 °C in ordinary atmosphere was demonstrated, although load reductions below 1.5 Log CFU/g were observed [15].

Chitosan coating produced different effects on lactic acid bacteria (Figure 1c), as both concentrations had the same inhibition pattern, reducing LAB load by at least 3 Log CFU/g compared to control at T7 and then keeping it below the detection limit (1 Log CFU/g) up to 28 days. A similar efficacy of chitosan on the reduction of LAB counts has been observed by other authors [23] on chicken breast fillets stored for 14 days at 4 °C in modified atmosphere. This result is noteworthy, as lactic acid bacteria constitute an important part of the natural and spoilage microbiota of meat in modified atmosphere or under-vacuum; therefore, their inhibition could have a direct impact on the product shelf life. 

Chitosan was particularly effective also in containing yeast growth during sample storage. This result highlights the activity of chitosan as an antimicrobial against eukaryotic and not only prokaryotic cells [24]. Coliforms were never detected in the samples analysed.

Therefore, chitosan was effective in containing or reducing the growth of all tested microorganisms. 

### 3.2. Chitosan Effect on Chemical and Physical Characteristics of Meat

With regard to the physicochemical characteristics (Table 1), the a_w_ values of the samples treated with chitosan, regardless of its concentration, substantially showed the same trend over time. Compared to control, the a_w_ of the treated samples was slightly higher, and the samples looked wetter. Other authors [17] highlighted an increased luminosity of chitosan-treated pork, probably due to protection from dehydration. 

Regarding pH, after a substantial reduction compared to control, due to the presence of acetic acid in chitosan solutions, the pH was kept constant in treated samples until the end of the experimental period. On the contrary, in the untreated control, this value increased with the days of storage, thus indicating a gradual alterative process of the protein fraction, with production of amines and NH_3_. Therefore, although the a_w_ values of the treated samples potentially allowed microbial growth, the substantial constancy of pH indicated a possible shelf life extension.

### 3.3. Sensory Analysis

Results of the sensory analysis are reported in Table 2. The panellists could not detect any taste difference between controls and samples treated with chitosan until day 14. As a comment, panellists were only able to detect higher odour intensity for chitosan samples. Results are in accordance with those on pork sausages [25], where chitosan did not change the sensory traits of the product. After days 21 and 28 of storage, no off-odours were perceived for the samples with chitosan, while some panellists detected pungent flesh odours for control samples, particularly after 28 days of refrigerated storage. These results are substantially in line with those obtained by other authors for pork loins treated with chitosan [17] and pork patties [15]. Both studies reported a positive effect of chitosan on acceptability of meat during refrigerated storage, with an improvement of the sensory characteristics of the samples. 

### 3.4. Rheological and Mechanical Properties of Chitosan Coating

Chitosan film was applied on Petri dishes and dried in the same conditions used for meat samples and eventually let equilibrate at a_w_ values of 0.984, corresponding to the lowest a_w_ value of chitosan-coated pork samples. After 7 days of equilibration, the film was in the liquid state. In order to study the rheological behaviour of the chitosan coating at a_w_ conditions of the fresh meat under examination, the chitosan solution was equilibrated at a_w_ of 0.984. In these conditions, the chitosan solution exhibited a liquid behaviour, and its dynamic viscosity was 229.4 ± 4.2 mPa·s. On the basis of this result, we can affirm that the chitosan coating was in the liquid state from at least the 7th day of the experiment; thus, it could not exert a significant impact on the mechanical properties of the meat samples.

Therefore, potential changes in the mechanical properties of the samples due to chitosan coating were further evaluated. Since meat samples show high variability in their mechanical properties, it was first necessary to standardize the support, and gelatine was chosen to mimic a protein-based support. Therefore, gelatine sheets of 2.5 g were dipped in 1% chitosan solution in the same experimental conditions as the pork samples and were subsequently submitted to a compression-shear-extrusion test. Before being analysed, the gelatine samples (untreated and chitosan coated) were balanced at a_w_ values of 0.984. The results are reported in Table 3, showing that chitosan had a negative effect on the compression modulus and on the maximum compression load. It can be inferred that chitosan coating causes structural modifications that could be explained by disaggregation of proteins due to the pH decrease to values below the isoelectric point of proteins.

Several authors have evaluated the physicochemical characteristics of films made of chitosan alone or, most commonly, added with plant extracts or plasticizers [26,27]. Nevertheless, a comparison of the data obtained is difficult because of the different chitosan employed (molecular weight, deacetylation grade), and most of all, because the samples were characterized as self-standing films and were not in contact with food products. In fact, when these films are put in contact with a food matrix with high a_w_, as in the experimental conditions adopted in this study, the chitosan films can adsorb water until they reach a viscous behaviour, and the physicochemical behaviour of the films themselves is no longer pertinent for the coated food characterization. In any case, it has to be considered that the antimicrobial activity of chitosan is expressed in an aqueous solution [28], but it is strongly reduced when the molecule is entrapped in insoluble films. In this respect, the application of chitosan as a viscous solution on the meat surface, as shown in this study, can be an important requirement to exert its antimicrobial potential [29], since the reactant mobility is much higher in the liquid state than in the solid state. 

## 4. Conclusions

In conclusion, the application of chitosan on vacuum-packed pork loins stored at 4 °C was effective in improving the safety of the product by containing *L. monocytogenes* growth. This is a valuable result because it was obtained by using a cocktail of strains, including strains isolated from meat products. Moreover, the chitosan coating showed a significant effect on the evolution of microbial populations associated with product shelf life. 

The beneficial effect of chitosan coating on pork shelf life was demonstrated also by sensory data and pH stability in treated samples. In our study, chitosan did not impair the sensory characteristics of the product; on the contrary, it increased its acceptability over time. Our results also demonstrated that chitosan is not a real film at the a_w_ conditions of fresh pork, but it behaves rather as a viscous solution and also protects meat from dehydration.

Further investigations are needed to optimise the system and also to determine the antimicrobial effect of chitosan on other pathogenic microorganisms that are able to contaminate fresh meat.

## Figures and Tables

**Table 1:** Water activity (a_w_) and pH values of vacuum-packed pork loin samples inoculated with *L. monocytogenes* and treated with chitosan 1% and 2%, stored at 4 °C for 28 days. Results are expressed as means ± standard deviations. Superscript letters indicate significant differences among the samples at the same storage time.

Time	a_w_			pH		
	Inoculated	Chitosan 1%	Chitosan 2%	Inoculated	Chitosan 1%	Chitosan 2%
0	0.988 ^a^ ± 0.001	0.988 ^a^ ± 0.001	0.988 ^a^ ± 0.001	5.90 ^a^ ± 0.03	5.90 ^a^ ± 0.03	5.90 ^a^ ± 0.03
1	0.987 ^a^ ± 0.001	0.987 ^a^ ± 0.001	0.986 ^a^ ± 0.002	5.83 ^a^ ± 0.02	5.65 ^b^ ± 0.01	5.68 ^b^ ± 0.03
7	0.982 ^b^ ± 0.002	0.988 ^a^ ± 0.001	0.984 ^b^ ± 0.001	5.83 ^a^ ± 0.04	5.55 ^c^ ± 0.04	5.58 ^c^ ± 0.02
14	0.984 ^b^ ± 0.001	0.984 ^b^ ± 0.002	0.986 ^a^ ± 0.001	5.94 ^a^ ± 0.03	5.63 ^b^ ± 0.03	5.66 ^b^ ± 0.03
21	0.990 ^a,c^ ± 0.002	0.986 ^a^ ± 0.002	0.986 ^a^ ± 0.002	6.00 ^a,d^ ± 0.03	5.62 ^b^ ± 0.02	5.64 ^b^ ± 0.01
28	0.988 ^a^ ± 0.001	0.992 ^c^ ± 0.001	0.992 ^c^ ± 0.001	6.20 ^d^ ± 0.02	5.63 ^b^ ± 0.02	5.60 ^b^ ± 0.02

**Table 2:** Results of the triangle test carried out on untreated pork loin samples (control) and samples treated with chitosan 1%, analysed after 1, 7, 14, 21, and 28 days of storage at 4 °C.

Chitosan vs. Control	Total Responses	Number of Correct Responses	Odour Intensity
Sampling Time			
Day 1	20	5	5/5 chitosan
Day 7	20	5	4/5 chitosan
Day 14	20	4	2/4 chitosan
Day 21 *	20	9	8/9 control
Day 28 *	20	16	16/16 ** control

* = sniffing test only; ** *p* < 0.001 significance level.

**Table 3:** Results of the compression-shear-extrusion analyses performed on gelatine alone (control) and on gelatine coated with 1% chitosan (chitosan-coated gelatine). Data on the same column with different letters are significantly different at a *p* < 0.05 level.

Sample	Max Compression Load (N)	Compression Modulus (N/mm)
Gelatine (control)	60.9 ± 6.0 ^a^	70.6 ± 3.8 ^a^
Chitosan-coated gelatine	54.6 ± 6.0 ^b^	60.7 ± 4.3 ^b^

## Supplementary Materials

The following are available online at http://www.mdpi.com/2304-8158/7/10/155/s1, Figure S1: Evolution of microbial parameters of vacuum-packed pork loin samples inoculated with *Listeria monocytogenes* (inoculated) and treated with chitosan 1% or acetic acid 1%, stored at 4°C for 14 days. (**a**) *Listeria monocytogenes*; (**b**) mesophilic aerobic count; (**c**) lactic acid bacteria; (**d**) yeasts.
