# Supplementary material for: Chitosan Coating Inhibits the Growth of Listeria monocytogenes and Extends the Shelf Life of Vacuum-Packed Pork Loins at 4 °C
Source: Foods. 2018 Sep 25;7(10):155. doi: 10.3390/foods7100155 (PMC6210955; doi:10.3390/foods7100155)
Supplement: Supplementary file 1 [file foods-07-00155-s001.pdf]

Supplementary Materials: Chitosan Coating Inhibits the Growth of *Listeria monocytogenes* and Extends the Shelf Life of Vacuum-Packed Pork Loins at 4 °C

Annalisa Serio, Clemencia Chaves-López, Giampiero Sacchetti, Chiara Rossi and Antonello Paparella *

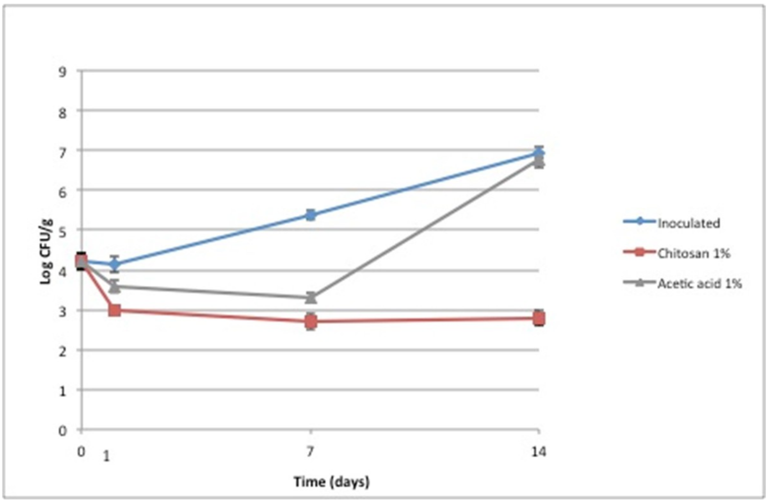

(a)

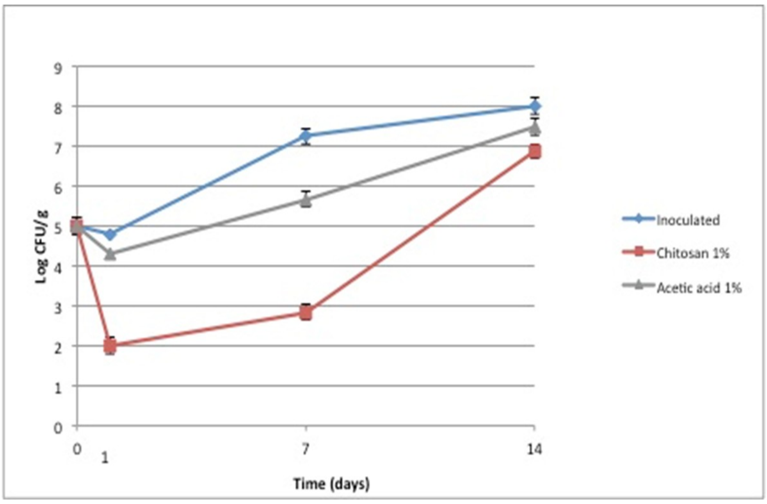

(b)

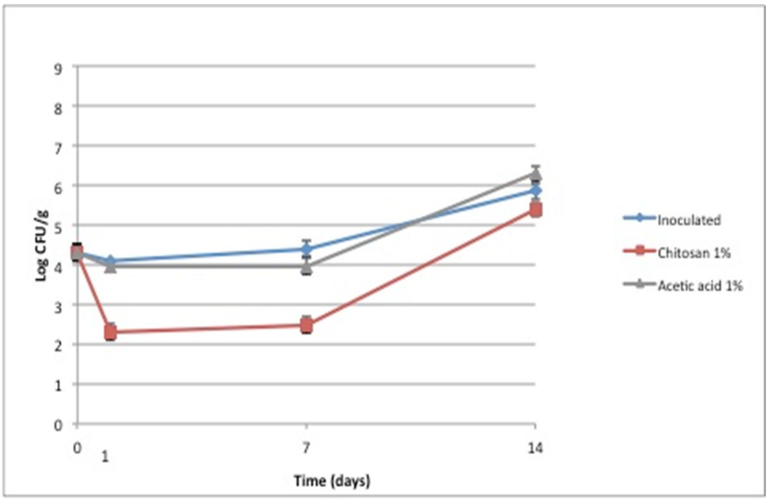

(c)

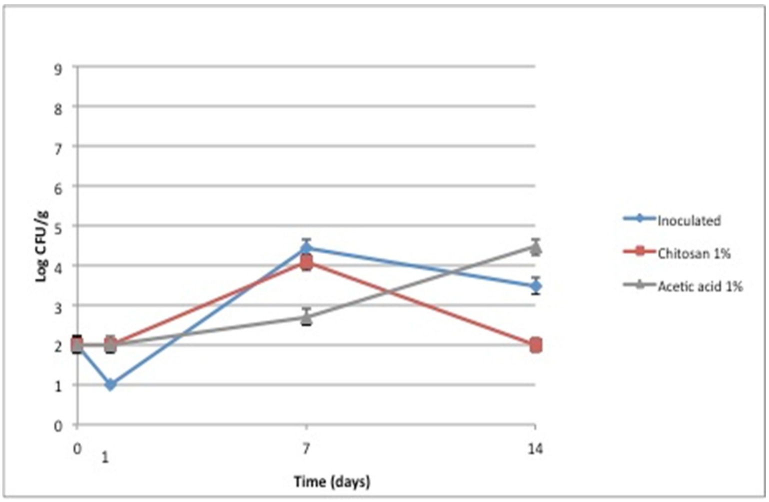

(d)

Figure S1. Evolution of microbial parameters of vacuum-packed pork loin samples inoculated with *Listeria monocytogenes* (inoculated) and treated with chitosan 1% or acetic acid 1%, stored at 4°C for 14 days. (**a**) *Listeria monocytogenes*; (**b**) mesophilic aerobic count; (**c**) lactic acid bacteria; (**d**) yeasts.
